# A PGRPLC1/Rel2-F axis controls *Anopheles gambiae* resistance to systemic infections with Gram-positive bacteria containing Lys-type peptidoglycan

**DOI:** 10.1371/journal.ppat.1013527

**Published:** 2025-09-19

**Authors:** Amani Audi, Suheir Zeineddine, Sana Jaber, Mike A. Osta

**Affiliations:** Department of Biology, American University of Beirut, Beirut, Lebanon; Imperial College London, UNITED KINGDOM OF GREAT BRITAIN AND NORTHERN IRELAND

## Abstract

In the Afrotropical malaria vector *Anopheles gambiae s.l.*, the Imd pathway plays pleiotropic roles in immunity, including resistance to malaria parasites, that are mediated by its NF-κB transcription factor Rel2. Rel2 exists as a full-length form (Rel2-F) containing the Rel-homology domain (RHD) and the C-terminal inhibitory ankyrin (Ank) and death domains (DD), and a shorter alternatively spliced form (Rel2-S) proposed to encode a constitutively active protein containing only the RHD. Despite its important roles in immunity, there are still multiple uncertainties concerning the identity and function of key components of the pathway as well as its overall contribution to mosquito resistance to systemic bacterial infections. Here, we show that Rel2 is critical for limiting the burden of Gram-negative and Gram-positive bacterial proliferation in *An. gambiae s.s.* after systemic infections and this function is attributed to the endoproteolytic activation of Rel2-F in the fat body but not to Rel2-S. Interestingly, while Rel2-F activation in the fat body regulates *Cecropin 1* and *Defensin 1* expression, its activation in the midgut after oral infections is dispensable for their regulation. We provide direct evidence that PGRPLC1 is necessary and sufficient for Rel2-F activation in the fat body in response to infections with Gram-positive bacteria containing Lysine-type peptidoglycan, however sensing of Gram-negative bacteria and Gram-positive bacilli containing DAP-type peptidoglycan is more complex and may be mediated by various PGRPLC isoforms, indicating that the mosquito Imd pathway integrates distinct receptor modules to sense Gram-positive and Gram-negative bacterial infections.

## Author summary

The Imd immune signaling pathway, broadly conserved in insects, is centered around antibacterial defense. In the malaria vector *Anopheles gambiae*, this pathway signals presumably through two isoforms of the NF-κB transcription factor Rel2, providing resistance also to malaria parasites. However, its molecular make-up suffers from several knowledge gaps, especially the relative contribution of each Rel2 isoform to antimicrobial defense. We provide evidence that during bacterial infections the mosquito Imd pathway signals only through the full-form Rel2 isoform, Rel2-F, whereas the short isoform Rel2-S does not seem to exhibit anti-bacterial roles. By probing Rel2-F cleavage dynamics in the fat body and midgut, we provide direct evidence on the identity of the pathway sensory receptors in the context of systemic and oral bacterial infections. Our findings contribute novel understanding about an immune pathway that constitutes an important intervention point for engineering malaria-resistant mosquitoes.

## Introduction

The Imd immune signaling pathway is broadly conserved in insects and regulates a potent antimicrobial effector response through the activation of NF-κB family transcription factors [1–6]. Comparative genomic analysis of the malaria vector *Anopheles gambiae* and *Drosophila melanogaster* identified the conserved components of the mosquito Imd pathway, including the NF-κB transcription factor Rel2 which is the orthologue of *Drosophila* Relish [7]. Rel2 pathway has received significant attention in *An. gambiae* due to its role in providing resistance to the human malaria parasite *P. falciparum* [8–11]. *An. gambiae* Rel2 exists as a full-length form (Rel2-F) containing the Rel-homology domain (RHD) and the C-terminal ankyrin (Ank) and death domains (DD), and a shorter form (Rel2-S) generated by alternative splicing containing only the RHD [12]. Rel2-S does not seem to harbor a unique sequence permitting its specific silencing by RNA interference (RNAi), unlike Rel2-F that can be silenced specifically by a double-stranded RNA (dsRNA) complementary to its C-terminal sequence encoding Ank and DD [12]. Hence, Rel2-S function has always been indirectly deduced by comparing the RNAi phenotypes obtained after silencing both forms simultaneously (using a dsRNA complementary to the RHD) to those obtained after silencing specifically Rel2-F. This approach identified Rel2-S as the culprit of the anti-*P. falciparum* defense mediated by the Rel2 pathway [9,11]. The anti-bacterial function of the Rel2 pathway has not been well characterized in mosquitoes as most studies have focused on the use of survival assays to gauge Rel2 function in immune defense [8,12,13]. Survival assays do not measure the host ability to limit the microbial burden in tissues but rather host tolerance, defined as the strategy by which the negative impact of infection on host fitness is reduced [14,15]. Importantly, the relative contributions of Rel2-F and Rel2-S to bacterial clearance during systemic infections are still unclear.

In *Drosophila*, PGRP-LC and PGRP-LE function as receptors of the Imd pathway [16–19] that recognize meso-diaminopimelic acid (DAP)-type peptidoglycan (PGN) of Gram-negative bacteria and several Gram-positive bacilli [20–22] to trigger Relish activation. *An. gambiae* genome lacks PGRPLE but contains a PGRPLC gene which encodes three main protein isoforms (PGRPLC1, -LC2 and -LC3) generated by alternative splicing [23], like its ortholog in *Drosophila* [24]. Functional studies by RNAi associated *An. gambiae* PGRPLC with defense against *Escherichia coli* (DAP-type PGN) and *Staphylococcus aureus* [Lysine (Lys)-type PGN] infections; all 3 isoforms were required for mosquito survival to *E. coli* infections, whereas only PGRPLC1 and PGRPLC3 isoforms were required for survival to *S. aureus* infections, with the latter exhibiting the strongest RNAi phenotype in both infections [23]. In contrast, reporter gene assays utilizing the *Cecropin 1* (*Cec1*) gene promoter identified PGRPLC1 as the main regulator of *Cec1* expression in the mosquito 4a3A cell line but not the other two isoforms [25]. Structural modeling suggested that all mosquito PGRPLC isoforms can bind either DAP- or Lys-type PGN [23], however, only binding to DAP-type PGN was confirmed biochemically [26]. Hence, there remains significant uncertainty as to which PGRPLC isoform functions as the *bona fide* receptor of the mosquito Imd pathway and whether this pathway is activated by DAP- or Lys-type PGN, or both. This uncertainty is driven by the fact that assigning immune roles to mosquito PGRPs has been largely based on survival assays and there have been no attempts to correlate the functions of these receptors to the endoproteolytic activation of Rel2. Additionally, a reliable readout for *An. gambiae* Imd pathway activation is still lacking, since the antimicrobial peptides (AMPs) Cec1, Defensin 1 and Gambicin, commonly used to gauge pathway activation are also regulated by Toll/Rel1 pathway [10].

Here, we show using a panel of bacteria that Rel2 pathway plays a central role in the clearance of Gram-negative and Gram-positive bacteria from the hemocoel during septic infections and provide direct evidence that this is driven by Rel2-F endoproteolytic activation in the fat body and not by Rel2-S. We also show that PGRPLC1 is necessary and sufficient for Rel2-F activation in the fat body in response to infections with Gram-positive bacteria containing Lys-type PGN. However, sensing of Gram-negative bacteria and Gram-positive bacilli containing DAP-type PGN is more complex and may be mediated by various PGRPLC isoforms.

## Results

### Mosquito Rel2 pathway provides resistance against systemic infections with Gram-positive and Gram-negative bacteria

**Fig 1:**
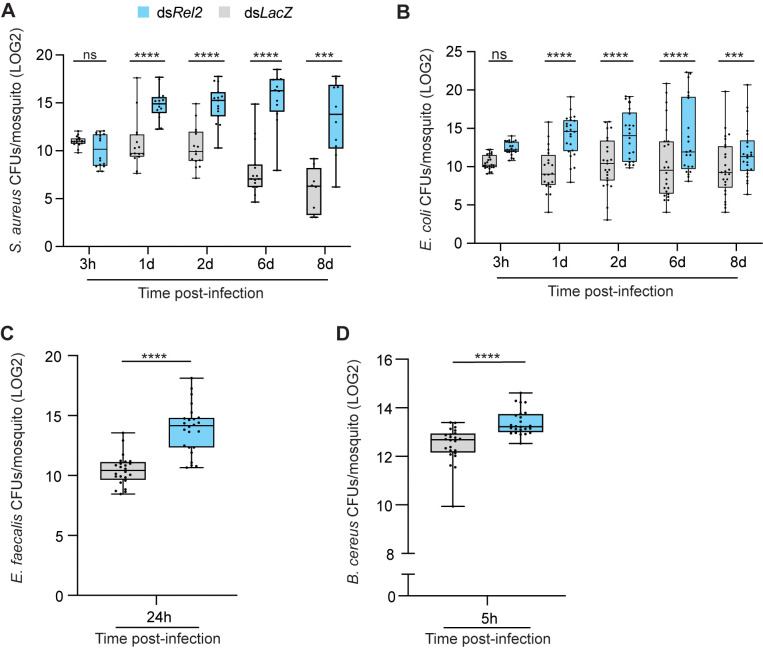
Rel2 pathway is essential for resisting systemic bacterial infections. Bacterial proliferation assays conducted on whole mosquitoes injected with (**A**) *S. aureus* (OD_600 _= 0.4), (**B**) *E. coli* (OD_600 _= 0.4), (**C**) *E. faecalis* (OD_600_ = 0.08), and (**D**) *B. cereus* (OD_600 _= 1). Batches of 6 whole mosquitoes each were homogenized in LB medium at the indicated time points after infection, and colony forming units (CFU) were scored on selective media. Data are presented as boxes and whiskers with medians and interquartile ranges. Each point on the scatter plot represents the mean LOG2 transformed CFU per mosquito in a given batch. Statistical analysis comparing bacterial proliferation in ds*Rel2* (blue bars) versus ds*LacZ* (grey bars) at every indicated time point was performed using Mann-Whitney test in (**A)** and (**D**), and two-tailed Welch’s t-test in (**B**) and (**C**). with *P*-values less than 0.05 considered significant. ****, *P* < 0.0001; *** *P* < 0.001. Data shown are from (A) 2, and (B-D) 3 independent experiments. ns, non-significant.

The contribution of Rel2 to anti-bacterial defense has been almost exclusively based on data obtained from mosquito survival assays [8,12,13], which measure host tolerance rather than resistance to infections. To determine the contribution of Rel2 to mosquito resistance to bacterial infections, adult female *An. gambiae* G3 mosquitoes were injected with double-stranded RNA complementary to *Rel2* (ds*Rel2*), then, 3 days later, challenged by intrathoracic injections of bacteria. Ds*Rel2* mosquitoes injected with *S. aureus* and *E. coli* exhibited significant bacterial proliferation in tissues at early and late time points post-infection (pi) compared to ds*LacZ* control (Fig 1A and 1B). Of note, *S. aureus* was rapidly cleared from ds*LacZ* mosquitoes, being barely detected by day 6 pi, whereas significant numbers remained in ds*Rel2* mosquitoes at day 8 pi. This compromised ability of ds*Rel2* mosquitoes to clear bacteria from the hemocoel was also observed with septic infections established with a virulent *Bacillus cereus* strain (Fig 1D) and *Enterococcus faecalis* (Fig 1C), indicating that the Rel2 pathway provides broad-spectrum protection against infections with Gram-positive and Gram-negative bacteria.

### Resistance to systemic infections requires Rel2-F endoproteolytic cleavage in the fat body

**Fig 2:**
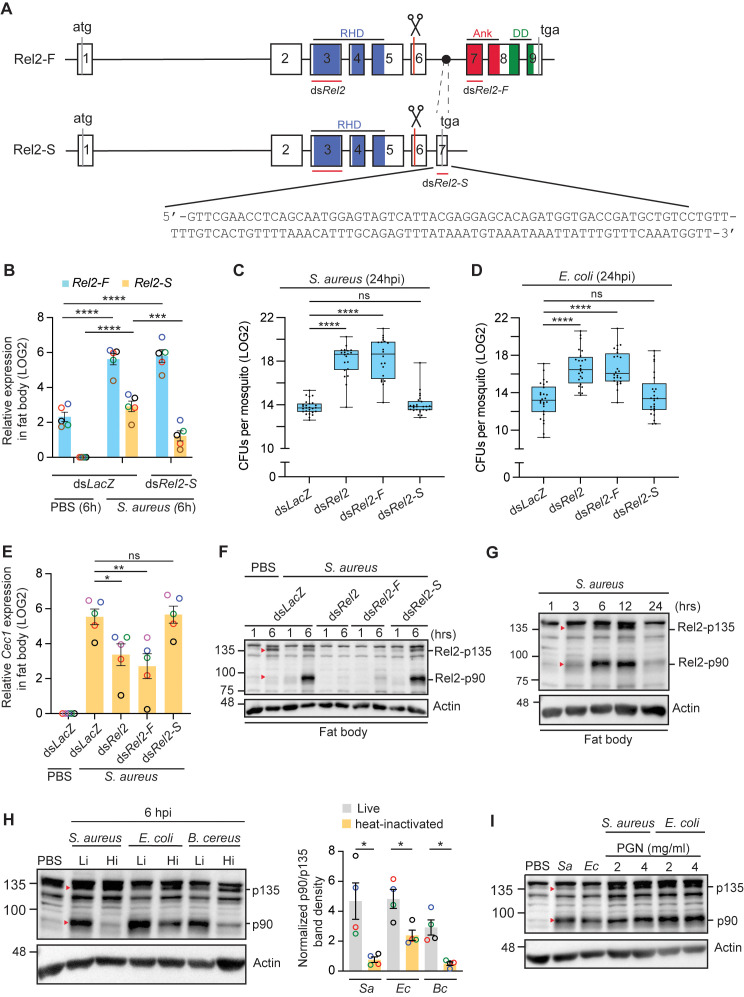
Rel2-F undergoes activation cleavage and is essential for resisting systemic bacterial infections. (**A**) Schematic drawing of the structural organization of Rel2-F and Rel2-S transcripts based on Vectorbase prediction and the previous study of Meister *et al.* [12]. Exons (boxes), introns (lines) and protein domains (in color) are shown. RHD, Rel homology domain; Ank, ankyrin domain: DD, death domain. Putative cleavage sites are indicated by scissors and regions targeted by the dsRNAs used to silence both forms (ds*Rel2*), the full form (ds*Rel2-F*) and the short form (ds*Rel2-S*) are indicated by red lines. The sequence of 130 nucleotides unique to Rel2-S exon 7 is shown. (**B**) QRT-PCR showing *Rel2-F* and *Rel2-S* relative expression in the fat body after *S. aureus* (OD_600_ = 3) and PBS (control) injections, and the specificity of ds*Rel2-S* silencing. Data are presented as mean ± SEM from 5 different biological experiments (shown in different colors). Statistical significance of the observed differences was calculated using one-way ANOVA followed by Dunnett’s multiple comparisons test. (**C-D**) Bacterial CFU counts in whole mosquitoes injected with (**C**) *S. aureus* (OD_600_ = 1) and (**D**) *E. coli* (OD_600_ = 1) in the indicated mosquito genotypes. Data from 3 independent experiments are presented as boxes and whiskers with medians and interquartile ranges. Each dot represents the LOG2 transformed mean CFU per mosquito in a given batch of 6 mosquitoes. Statistical analysis was performed using Kruskal-Wallis test followed by Dunn’s multiple comparisons test. (**E**) *Cecropin 1* (*Cec1*) expression measured by qRT-PCR in the fat body of the indicated mosquito genotypes after injection with *S. aureus* (OD_600_ = 3) or sterile PBS (control). Data are presented as mean ± SEM from 5 independent experiments (shown in different colors). Statistical analysis is as in panel B. (**F-G**) Western blots showing Rel2-F endoproteolytic cleavage in the fat body after *S. aureus* (OD_600_ = 3) injection of (**F**) dsRNA-treated mosquitoes and (**G**) wildtypes. β-actin was used as loading control. Each lane contains fat body extracts equivalent to 2 mosquito abdomens (excluding guts and ovaries). Each image is representative of 2 independent experiments. (**H-I**) Western blots showing Rel2-F cleavage in fat body of mosquitoes at 6 hpi with (**H**) live (Li) or heat-inactivated (Hi) *S. aureus* (OD_600_ = 3), *E. coli* (OD_600_ = 3) and *B. cereus* (OD_600_ = 2) and (**I**) with live *S. aureus* (*Sa*), *E. coli* (*Ec*) at the same OD as in panel H, and with purified polymeric *S. aureus* and *E. coli* peptidoglycan (PGN). The graph bar in panel H represents the normalized p90/p135 band density with respect to β-actin (loading control) from 4 independent biological experiments. Statistical analysis was performed using two-tailed Welch’s t-test. The figure in panel I is representative of at least 2 independent biological experiments. Red triangles correspond to Rel2-p135 and Rel2-p90. ****, *P* < 0.0001; ***, *P* < 0.001; **, *P* < 0.01; *, *P* < 0.05. ns, non-significant.

Ds*Rel2* is complementary to a region in the RHD that is shared by Rel2-F and Rel2-S, therefore it silences both transcripts (Fig 2A and [12]). To investigate which of these two transcripts is the main contributor to mosquito anti-bacterial defense, we opted to specifically silence each of these transcripts independently. To specifically silence Rel2-F, we utilized ds*Rel2-F* that targets a sequence in the region encoding the ankyrin domain (Fig 2A), as previously described [12]. However, the specific silencing of Rel2-S has not been described so far, as it is thought to be identical in sequence to Rel2-F, with the exception that it lacks the sequences encoding the ankyrin and death domains [12]. Since we could not find any nucleotide sequence for Rel2-S in the literature nor in the databases, we performed 3′ RACE to try to identify any unique sequence in the 3′ end of Rel2-S that could be leveraged for the specific silencing of this transcript; if there exist any significant sequence divergence between both transcripts, it is expected to be towards the 3′ end (Fig 2A). Interestingly, we identified a contiguous sequence of 130 nucleotides at the distal 3′ end of Rel2-S that is unique to this transcript, of which 46 nucleotides are predicted to be coding while the rest are in 3′ UTR (Figs 2A and S1). We designed ds*Rel2-S* that is complementary to this region (Fig 2A) to silence specifically Rel2-S and showed that ds*Rel2-S* indeed silences Rel2-S but not Rel2-F (Fig 2B). We also showed that both transcripts are significantly upregulated in the fat body at 6 hours (h) pi with *S. aureus* (Fig 2B). Interestingly, ds*Rel2* and ds*Rel2-F* mosquitoes exhibited similar compromised abilities to clear *S. aureus* (Fig 2C) and *E. coli* (Fig 2D) infections, whereas ds*Rel2-S* mosquitoes cleared the bacteria as efficiently as the ds*LacZ* control group. Additionally, at 6 hpi with *S. aureus*, ds*Rel2-S* mosquitoes showed normal *Cec1* (Fig 2E) and *Defensin 1* (*Def1*) (S2 Fig) expression in the fat body, whereas *Cec1* and *Def1* expression was significantly reduced in ds*Rel2* and ds*Rel2-F* mosquitoes. Altogether, these results suggest that Rel2-S does not play a role in defense against septic bacterial infections.

It remains unclear whether activation of mosquito Rel2-F requires its endoproteolytic cleavage to release the inhibitory ankyrin and death domains, like its *Drosophila* orthologue Relish [27,28]. Using a Rel2-specific antibody raised against the RHD, we show that *S. aureus* infection triggered Rel2-F endoproteolytic cleavage in the fat body resulting in a 90 kDa (Rel2-p90) cleaved product corresponding in size to Rel2-F lacking the ankyrin and death domains (Fig 2F). Treating mosquitoes with ds*Rel2* or ds*Rel2-F* strongly reduced Rel2-F full (p135) and cleaved (p90) forms in the fat body to similar extents (Fig 2F). Rel2-S is expected to encode a constitutively active transcription factor of similar size to the cleaved Rel2-F p90 form, since it lacks the ankyrin and death domains. However, silencing *Rel2-S* did not reduce p90 protein levels in the fat body (Fig 2F), indicating that Rel2-S protein is not expressed in the fat body during bacterial infections, in agreement with its RNAi phenotypes that show no role in bacterial clearance nor in regulating *Cec1* and *Def1* expression. Rel2-F cleavage in the fat body peaks between 6 and 12 hpi, returning to basal levels by 24 hpi (Fig 2G), indicating that Rel2 activation is subject to tight temporal regulation. Rel2-F cleavage was efficiently induced by bacteria containing Lys-type (*S. aureus*) and DAP-type PGN (*E. coli*, *B. cereus*) (Fig 2H). Injecting heat-inactivated bacteria dramatically reduced Rel2-F cleavage which was almost abolished with heat-inactivated *S. aureus* and *B. cereus* (Fig 2H), indicating that bacterial proliferation is likely important for efficient Rel2-F activation. Of note, although septic infection is clearly upregulating Rel2-F protein, the increase in p135 (full form) levels is always modest because it is rapidly cleaved to p90 (Fig 2F and 2G). Injecting pure polymeric *S. aureus* and *E. coli* PGN efficiently triggered Rel2-F cleavage indicating that PGN is indeed the inducer of the Imd pathway (Fig 2I).

### PGRPLC1 induces Rel2-F cleavage in response to infections with Gram-positive bacteria containing Lysine-type peptidoglycan

**Fig 3:**
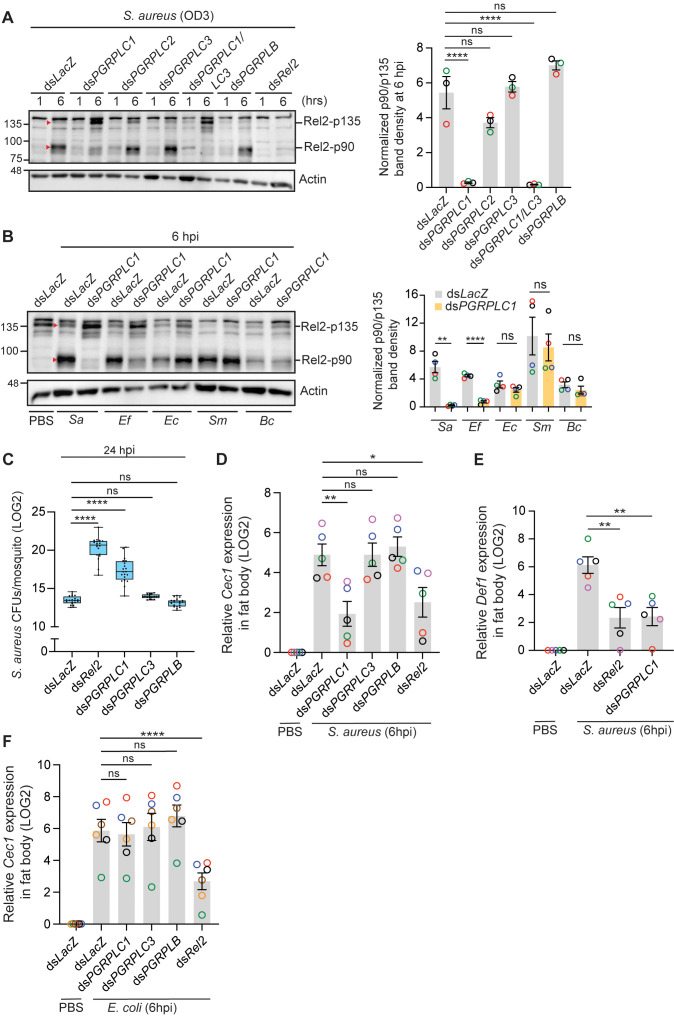
PGRPLC1 controls Rel2-F activation after infections with Lys-type PGN-containing bacteria. (**A-B**) Western blots showing Rel2-F cleavage in the indicated gene knockdowns after infection with (**A**) *S. aureus* (OD_600_ = 3), (**B**) *S. aureus* (*Sa*, OD_600_ = 3), *E. faecalis* (*Ef*, OD_600_ = 3), *E. coli* (*Ec*, OD_600_ = 3), *S. marcescens* (*Sm*, OD_600_ = 1) and *B. cereus* (*Bc*, OD_600_ = 2). Each lane contains fat body extracts equivalent to 2 mosquito abdomens (excluding gut and ovaries). The graph bars represent the normalized p90/p135 band density at the 6-hour time point with respect to β-actin (loading control) from 3 and 4 independent experiments (shown in different colors) in panels A and B, respectively. Statistical analysis in panel A was performed using One-way ANOVA followed by Dunnett’s multiple comparisons test and in panel B using the two-tailed Welch’s t-test by comparing the means of the two groups in each infection. Red triangles correspond to Rel2-p135 and Rel2-p90. (**C**) Bacterial CFU counts in whole mosquito homogenates injected with *S. aureus* (OD_600_ = 1). Data from 2 independent experiments are presented as boxes and whiskers with medians and interquartile ranges. Each dot represents the LOG2 transformed mean CFU per mosquito in a given batch of 6 mosquitoes. Statistical analysis was performed using One-way ANOVA followed by Dunnett’s multiple comparisons test. (**D-F**) QRT-PCR analysis of (**D** and **F**) *Cec1* and (**E**) *Def1* expression measured in the fat body of the indicated mosquito genotypes after injection with *S. aureus* (OD_600_ = 3) or *E. coli* (OD_600_ = 3). Data are presented as mean ± SEM from 5, 5 and 6 independent experiments (shown in different colors), respectively. Statistical analysis was performed using One-way ANOVA followed by Dunnett’s multiple comparisons test. ****, *P* < 0.0001; *** *P* < 0.001; ** *P* < 0.01; * *P* < 0.05. ns, non-significant.

Functional genetic studies and gene reporter assays in *An. gambiae* mosquitoes and cell lines, respectively, proposed PGRPLC as the putative receptor of the Imd/Rel2 pathway in response to infections with Gram-positive and Gram-negative bacteria, but PGRPLC function has not been directly linked to Rel2 activation [23,25]. Furthermore, it remains controversial which of the three main PGRPLC isoforms (LC1, LC2 or LC3) is the sensor of Lysine-type PGN of Gram-positive bacteria and DAP-type PGN of Gram-negative bacteria and some Gram-positive bacilli, as mosquito survival assays implicated all 3 isoforms to different extents in defense against *S. aureus* and *E. coli* infections, with PGRPLC3 exhibiting the strongest RNAi phenotype [23]. Also, *in silico* structural modeling suggested that all isoforms can bind both PGN types [23], and pull-down assays showed that recombinant PGRPLC1 and PGRPLC3 ectodomains interact with DAP-type PGN [26]. Here, we leveraged Rel2-F activation cleavage in the fat body as a readout to investigate which of the PGRPLC isoforms is responsible for sensing infections with Lys- and DAP-type PGN-containing bacteria. We used the same dsRNA templates described by Rodgers *et al*. [26] to silence the different isoforms. Silencing *PGRPLC1*, but not *PGRPLC2* or *PGRPLC3*, almost abolished Rel2-F cleavage at 6 hpi with *S. aureus*, with concomitant enrichment of its p135 full-form (Fig 3A). Co-silencing *PGRPLC1* and *PGRPLC3*, which was previously proposed to be the main isoform involved in anti-bacterial defense [23], gave the same phenotype as *PGRPLC1* silencing (Fig 3A). *PGRPLC1* kd inhibited significantly Rel2-F cleavage in the fat body after infections with *S. aureus* and *E. faecalis* (contain Lys-type PGN) but not with *E. coli*, *S. marcescens*, or *B. cereus*, all of which contain DAP-type PGN (Fig 3B). Furthermore, *PGRPLC1* kd reduced significantly mosquito resistance to *S. aureus* infections (Fig 3C) and *S. aureus*-induced *Cec1* (Fig 3D) and *Def1* (Fig 3E) upregulation to similar levels as ds*Rel2*, whereas *PGRPLC3* kd exhibited similar RNAi phenotypes as ds*LacZ* control (Fig 3C and 3D). Neither *PGRPLC1* nor *PGRPLC3* kd, however, reduced *Cec1* expression after *E. coli* infections (Fig 3F). Altogether, these results indicate that PGRPLC1 is the *bona fide* receptor of the Imd pathway in response to infections with Gram-positive bacteria containing Lys-type PGN.

**Fig 4:**
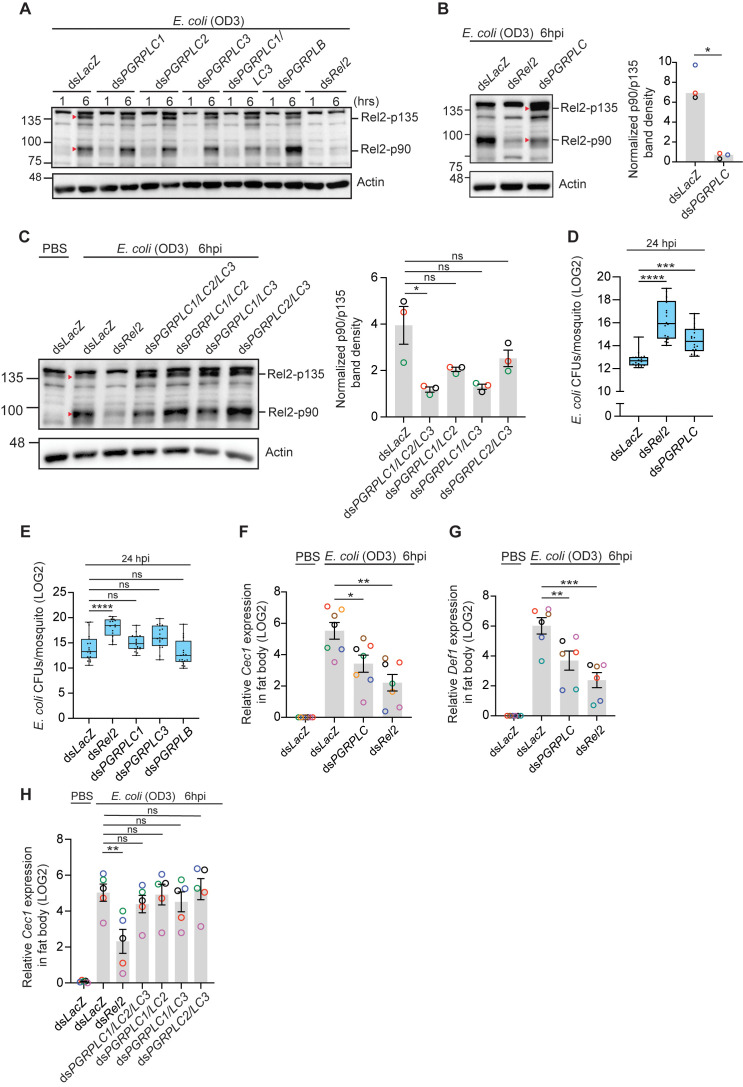
Rel2-F activation cleavage in response to *E. coli* infections requires PGRPLC. (**A-C**) Western blots showing Rel2-F cleavage in the indicated gene knockdowns and time post-infection with *E. coli* (OD_600_ = 3). β-actin was used as loading control. Each lane contains fat body extracts equivalent to 2 mosquito abdomens (excluding gut and ovaries). Each image is representative of (A) 2, (B) 3 and (C) 3 independent experiments. The graph bars in panels B and C represent the normalized p90/p135 band density. Statistical analysis was performed using the two-tailed Welch’s t-test in panel B and Kruskal-Wallis test followed by Dunn’s multiple comparisons test in panel C. *, *P* < 0.05. Red triangles correspond to Rel2-p135 and Rel2-p90. (**D-E**) Bacterial CFU counts in whole mosquito homogenates in the indicated gene knockdowns after injection with *E. coli* (OD_600_ = 1). Data from 2 independent experiments are presented as boxes and whiskers with medians and interquartile ranges. Each dot represents the LOG2 transformed mean CFU per mosquito in a given batch of 6 mosquitoes. Statistical analysis was performed using Kruskal-Wallis test followed by Dunn’s multiple comparisons test. (**F-H**) QRT-PCR analysis of (**F** and **H**) *Cecropin 1* (Cec1) and (**G**) *Defensin 1* (Def1) expression in the fat body of the indicated mosquito genotypes at 6 hpi with *E. coli* (OD_600_ = 3) or injection of sterile PBS (control). Data are presented as mean ± SEM from 7, 6 and 5 independent experiments (shown in different colors) in panels F, G and H, respectively. Statistical analysis was performed using One-way ANOVA followed by Dunnett’s multiple comparison test. ****, *P* < 0.0001; ***, *P* < 0.001; **, *P* < 0.01; *, *P* < 0.05. ns, non-significant.

The fact that Rel2-F cleavage was not altered in ds*PGRPLC1* mosquitoes infected with bacteria containing DAP-type PGN prompted us to test whether the other two isoforms function in concert to sense these bacterial infections. Silencing the PGRPLC isoforms individually or in different pairwise combinations did not inhibit Rel2-F cleavage in response to *E. coli* infections (Fig 4A and 4C). We then tested whether silencing all isoforms simultaneously by injecting a dsRNA mixture containing ds*PGRPLC1*, ds*PGRPLC2* and ds*PGRPLC3* or a dsRNA that targets an exon common to the PGRP domains of all possible PGRPLC isoforms (ds*PGRPLC*, [29]), would inhibit Rel2-F cleavage after *E. coli* infections. Interestingly, whereas Rel2-F cleavage was strongly inhibited in ds*PGRPLC* mosquitoes concomitant with a clear enrichment in p135 (Fig 4B), it was relatively moderately inhibited in ds*PGRPLC1*/*LC2*/*LC3* mosquitoes (Fig 4C). Ds*PGRPLC* mosquitoes exhibited significant proliferation of *E. coli* (Fig 4D) which was not observed in ds*PGRPLC1* or ds*PGRPLC3* mosquitoes (Fig 4E). Also, *E. coli*-induced *Cec1* and *Def1* upregulation were systematically reduced in ds*PGRPLC* compared to ds*LacZ* mosquitoes in all experiments (Fig 4F and 4G). In contrast, *Cec1* expression in *E. coli*-infected mosquitoes treated with different dsRNA pairwise combinations of the main PGRPLC isoforms or with the ds*PGRPLC1*/*LC2*/*LC3* mixture was similar to that in ds*LacZ* controls (Fig 4H). Altogether, these results suggest that PGRPLC is involved in sensing DAP-type PGN, however several isoforms seem to be redundantly involved in that process. Among all tested PGRP isoforms, PGRPLC1 and PGRPLC3 exhibited excellent silencing efficiencies in single gene knockdowns by RNAi reaching ~80% and ~90%, respectively, whereas PGRPLC2 showed a more modest silencing efficiency ranging between 25 and 40% (S3A Fig). The ds*PGRPLC1*/*LC2*/*LC3* mixture exhibited slightly better gene silencing efficiencies of the 3 main PGRPLC isoforms than ds*PGRPLC* (S3B Fig).

### PGRPLB does not regulate the mosquito Imd pathway in the fat body during septic infections

*Drosophila* PGRP-LB negatively regulates the Imd pathway in systemic and local gut infections with Gram-negative bacteria [30–32]. In *An. coluzzii*, *PGRPLB* silencing enhanced *Cec1* expression in the fat body of naïve mosquitoes at 6 days post-silencing and of blood-fed mosquitoes at 3 days post-blood ingestion, suggesting that it may play a similar role to its *Drosophila* ortholog [29], however, its role in regulating the Imd pathway during systemic bacterial infections has not been investigated. Here, we show that *PGRPLB* silencing did not enhance Rel2-F activation cleavage during systemic infections with *S. aureus* (Fig 3A) and *E. coli* (Fig 4A); although Rel2 p90 was apparently enriched in *E. coli* infections, this pattern was not systematically observed (S4C Fig). These results agree with the lack of effect on *Cec1* regulation in *PGRPLB* silenced mosquitoes at 6 hpi with *S. aureus* (Fig 3D) and *E. coli* (Fig 3F), and the fact that ds*PGRPLB* mosquitoes did not exhibit enhanced clearance of *S. aureus* (Fig 3C) and *E. coli* (Fig 4E) at 24 hpi. We then asked whether this absence of regulation over *Cec1* is probably due to the early time point (6 hpi) at which it was assessed, especially that PGRPLB is a negative regulator, and its activity may be more pronounced at later time points to prevent extended activation of the Imd/Rel2 pathway. However, *PGRPLB* kd did not alter *Cec1* expression in the fat body at 72 hpi with *S. aureus* (S4A Fig), nor did it prolong Rel2-F activation cleavage at 24 hpi with *S. aureus* (S4B Fig) and *E. coli* (S4C Fig). The mosquito midgut microbiota undergoes dramatic proliferation after a blood meal [23,33], which prompted us to test whether PGRPLB could be negatively regulating the Imd pathway in the fat body in response to PGN fragments that might leak from the distended midgut after blood feeding. Surprisingly, Rel2-F expression in the fat body at 6, 15 and 24 hours after a blood meal was similar to that in sugar-fed mosquitoes and the cleaved p90 form was barely detected only at 6h post-blood feeding (S5A-S5C Fig). *PGRPLB* silencing did not enhance Rel2-F expression nor cleavage after blood feeding (S5B and S5C Fig). Again, we did not detect a protein product for Rel2-S in the fat body of blood-fed mosquitoes. Altogether, these results suggest that the Imd pathway does not seem to be induced in the fat body after a blood meal.

### Rel2-F cleavage in the midgut after oral infections requires PGRPLC

**Fig 5:**
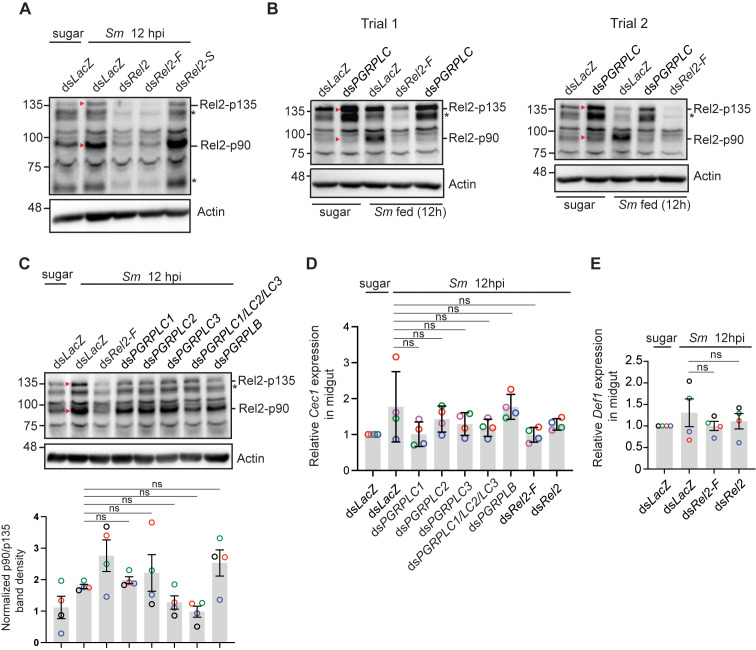
PGRPLC controls Rel2-F cleavage in the midgut after oral infections. (**A-C**) Western blots showing Rel2-F cleavage in the midgut of the indicated mosquito genotypes after feeding on sugar or on *S. marcescens* (*Sm*, OD_600_ = 15) for 12 hours. β-actin was used as loading control. Each lane contains extracts from 5 mosquito midguts (including cardia, anterior and posterior midguts). The images shown are representative of 2, 2 and 4 independent experiments in panels A, B (both shown) and C, respectively. The bar graph under the western blot in panel C represents the normalized p90/p135 band density with respect to β-actin (loading control). The bars in the graph align with their corresponding lanes in the western blot above. Data are presented as mean ± SEM from 4 independent experiments (shown in different colors). Statistical analysis was performed using using One-way ANOVA followed by Dunnett’s multiple comparisons test. Red triangles indicate Rel2-p135 and Rel2-p90. Asterisks (*) refer to Rel2 bands generated likely by enzymatic activity in the midgut during extraction. (**D**) *Cec1* and (**E**) *Def1* expression measured by qRT-PCR in the midguts (including cardia, anterior and posterior midguts) of the indicated mosquito genotypes after oral infections with *S. marcescens* (OD_600_ = 15). Data are presented as mean ± SEM from 4 independent experiments (shown in different colors). Statistical analysis was performed using One-way ANOVA followed by Dunnett’s multiple comparisons test. ns, non-significant.

The Imd/Rel2 pathway is also active in the *Anopheles* midgut where it provides resistance to invading *Plasmodium* parasites [8–11], controls gut microbiota proliferation [23] and regulates peritrophic matrix formation [34]. To determine whether Rel2-F exhibits similar activation cleavage dynamics in the midgut as in the fat body, we first fed mosquitoes on *S. marcescens* containing sugar pads and monitored Rel2-F cleavage in mosquito midguts dissected at 12 hours post-continuous feeding on the bacteria. The p90 form was clearly more pronounced in the midguts of *S. marcescens*-fed ds*LacZ* mosquitoes than in the sugar-fed groups which exhibited basal levels of p90, probably triggered by the microbiota (Fig 5A-5C). Both, the p90 and p135 forms of Rel2 were strongly reduced in *S. marcescens*-fed ds*Rel2* and ds*Rel2-F* but not in ds*Rel2-S* mosquitoes (Fig 5A), recapitulating the results obtained in the fat body that Rel2-S is not translated under the tested experimental conditions. The individual silencing of the 3 main PGRPLC isoforms did not inhibit Rel2-F cleavage in response to *S. marcescens* oral infections (Fig 5C) nor did it alter *Cec1* expression at 12 hpi with *S. marcescens* (Fig 5D). Interestingly, injecting mosquitoes with ds*PGRPLC* (Fig 5B) but not the ds*PGRPLC1*/*LC2*/*LC3* mixture (Fig 5C) strongly inhibited Rel2-F cleavage after oral *S. marcescens* infections with concomitant enrichment of the full form, indicating that PGRPLC controls REL2-F activation cleavage in the gut after sensing Gram-negative bacteria. Even in naïve, sugar-fed mosquitoes, ds*PGRPLC* inhibited the basal cleavage of Rel2-F probably triggered by the microbiota, as shown by the enrichment of Rel2-p135 (Fig 5B). In the midgut, the Rel2-F full form appears as two bands; the typical 135 kDa band that is also detected in the fat body and another band at ~125 kDa that is only detected in midgut extracts. This lower band could be generated either by cleavage of p135 by gut enzymes during extraction or by tissue-specific alternative splicing at the 5′ end of Rel2-F which was previously shown to contain two alternative 5′ exons [12]. *Cec1* and *Def1* expression were not significantly upregulated in the midgut after *S. marcescens* infections and their expression does not appear to be regulated by Rel2 (Fig 5D and 5E). This contrasts with their strong upregulation in the fat body after systemic infections in a Rel2-dependent manner (Figs 2E, S2, 3D and 3E).

## Discussion

The Imd pathway is a central arm of insect antimicrobial immunity that deals primarily with bacterial infections [35,36]. Here, we show that the *An. gambiae* Imd/Rel2 pathway is a central player in resistance to bacterial infections since Rel2 silencing compromised the mosquito ability to clear systemic infections with both Gram-positive and Gram-negative bacteria. Rel2-F mediates the anti-bacterial resistance and undergoes endoproteolytic cleavage in the fat body in response to systemic infections that peaked at 6 hpi, returning to basal levels at 24 hpi, suggesting that the pathway is tightly regulated. Rapid activation of the Imd pathway has been also described in *Drosophila* [27,37]. It has been proposed that Relish is rapidly activated to control fast-replicating microbes like bacteria in the fly tissues [38,39], in contrast to the anti-fungal Toll pathway that is activated within hours with AMP expression peaking at 24 hpi and persisting for days [40]. Our work also suggests that Rel2-S, a minor splice variant of Rel2, does not seem to be translated neither in the fat body nor in the midgut under the experimental conditions tested herein. This explains why Rel2-S silencing did not alter mosquito resistance to bacterial infections nor antimicrobial peptide expression. Indeed many genes generate splice variants that are not translated and appear to be generated from noise in the splicing machinery [41,42]. Nevertheless, this result was surprising since Rel2-S has been associated with defense against *P. falciparum* in *An. gambiae* mosquitoes [9,11]. This role for Rel2-S has been indirectly deduced by comparing the RNAi phenotypes of ds*Rel2* (that silences both Rel2-S and Rel2-F) and ds*Rel2-F* [9,11], since, prior to our work, there has been no attempt to specifically silence *Rel2-S*. Since a functional role for Rel2-S has been proposed, so far, only in the context of *Plasmodium* infections, it remains possible that Rel2-S is transiently expressed in the midgut during blood feeding to regulate the microbiota proliferation and/or certain aspects of the midgut physiology, which we could not assess by western blot due to technical hurdles associated with the masking effect of blood bolus proteins. Future work should assess the relative contributions of Rel2-F and Rel2-S to regulating the microbiota and midgut physiology in response to blood feeding. Interestingly, blood feeding did not trigger Rel2-F cleavage in the fat body despite the blood meal-induced dramatic proliferation of the midgut microbiota [23,33], suggesting that either the distended midgut does not leak during a blood meal or that PGRP amidases neutralize the immunostimulatory PGN in the midgut lumen as shown in *Drosophila* [30,31,43,44].

We show that PGRPLC1 is the *bona fide* receptor of the Imd pathway in the context of infections with Gram-positive bacteria containing Lys-type PGN, since its knockdown abolished Rel2-F activation cleavage, reduced *Cec1* and *Def1* upregulation in the fat body and triggered significant bacterial proliferation in the hemolymph after *S. aureus* infections. Our results are in line with a previous study showing that PGRPLC1 is the main regulator of *Cec1* promoter in the *An. gambiae* 4a3A cell line [25]. In contrast, Meister *et al* have previously shown that silencing the 3 main PGRPLC isoforms simultaneously and not PGRPLC1 alone was required to inhibit *Cec1* upregulation [23]. A plausible explanation for this discrepancy is that in their study, *Cec1* expression was gauged in whole mosquito extracts and at 3 hpi with *S. aureus* [23], whereas we scored *Cec1* expression at 6 hpi in the fat body. At 3 hpi, *Cec1* expression in the fat body might not have peaked enough to allow the detection of a clear phenotype with *PGRPLC1* kd, in addition to masking effects from the relatively high, constitutive expression levels of *Cec1* in the midgut. Rel2-F was also cleaved in the fat body and midgut after infections with bacteria containing DAP-type PGN in a PGRPLC-dependent manner, however, sensing of DAP-type PGN seems to be goverened by multiple PGRPLC isoforms. This is supported by a previous work showing that the recombinant ectodomains of PGRPLC1 and PGRPLC3 co-precipitated with polymeric DAP-type PGN of *E. coli*, whereas PGRPLC2 and PGRPLC3 bound to the *E. coli* peptidoglycan monomer, tracheal cytotoxin [26], inferring that all isoforms might be involved in sensing Gram-negative bacteria. Furthermore, the fact that ds*PGRPLC* was relatively slightly less efficient in silencing all 3 main isoforms than the ds*PGRPLC1*/*LC2*/*LC3* mixture, yet it was more efficient in inhibiting Rel2-F cleavage in the fat body and midgut as well as the upregulation of *Cec1* and *Def1* in the fat body, suggests that additional PGRPLC isoforms with hybrid domains may also be involved in sensing DAP-type PGN. This relaxed mode of recognition may not be suprising since the majority of the mosquito gut microbiota are Gram-negative bacteria, which predicts that infections triggered by crossing the midgut will likely be inflicted by these Gram-types. The PGRPLC gene was previously reported to harbor a complex architecture encoding, in addition to the 3 major isoforms, also transcripts with hybrid PGRP domains, such as an LC2/LC3 hybrid [23]. This is possible because the PGRP domain of each of the major isoforms is encoded by one exon shared by all isoforms and two variable exons [23]. Our results suggest that ds*PGRPLC*, which targets the exon common to all isoforms [23], gives better RNAi phenotypes than the ds*PGRPLC1*/*LC2*/*LC3* mixture, probably by sufficiently inhibiting all possible transcripts, whereas PGRPLCs with hybrid domains may escape, at least partially, silencing by the isoform-specific dsRNA mixture. A more thorough sequencing of the PGRPLC gene transcripts will be required to clarify this discrepancy.

In the midgut, although Rel2-F is cleaved following oral infections with *S. marcescens* in a PGRPLC-dependent manner, it does not seem to regulate *Cec1* nor *Def1* expression. In fact, oral infections did not upregulate *Cec1* nor *Def1* in the midgut, suggesting that these, and possibly other, AMPs are constitutively expressed at sufficient levels and that higher levels may damage the microbiota. However, since our midgut samples included the cardia, anterior and posterior midguts, we cannot exclude that antimicrobial peptide expression in the midgut may be driven by Rel2-F in a more compartmentalized manner. Since Rel2-F regulates AMP expression in the fat body during systemic infections but not in the midgut during oral infections, raises the question whether the transcriptional programs triggered by the Imd/Rel2 pathway in these two immune tissues are only partially overlapping. Tissue-specific transcriptomic analysis in *Rel2* kd mosquitoes will be required to address this point.

Studies in *D. melanogaster* [30,31] and *An. coluzzii* [29] identified a role for PGRPLB in negatively regulating the Imd pathway, however, in the latter species, this role was investigated only in the context of naïve and blood-fed mosquitoes. It is intriguing that we have not observed any effect of *PGRPLB* kd on *Cec1* expression nor on the kinetics of Rel2-F activation cleavage in the fat body and midgut after bacterial infections, despite the fact that PGRPLB is expressed in both tissues according to our previous RNAseq data [45]. Gendrin *et al* showed that PGRPLB silencing in naïve *An. coluzzii* mosquitoes enhanced *Cec1* expression in the fat body at 6 days post-ds*PGRPLB* injection [29]. The latest time point at which we measured *Cec1* expression in the fat body of bacteria-infected ds*PGRPLB* mosquitoes is 3 days pi. At this time point, mosquitoes still harbour significant numbers of bacteria, according to our time-course bacterial proliferation assays, which we speculate may overwhelm PGRPLB activity. PGRPLB function may be more important at later time points to shut down the response as bacterial numbers decrease in the tissues. Rel2-F was expressed at basal levels in the fat body until 24 hours after blood feeding with no evidence of cleavage taking place, and this pattern did not change after silencing *PGRPLB*. Our results agree with a previous report showing that PGRPLB does not regulate *Cec1* expression in the fat body of *An. coluzzii* at early time points post blood-feeding [29]. Although a blood meal triggers dramatic proliferation of the mosquito midgut microbiota [23,33], it would be premature to conclude at this stage that PGRPLB does not play a role in preventing the systemic activation of the Imd pathway in response to midgut bacterial proliferation, since we have not measured Rel2-F cleavage and *Cec1* expression in the fat body after feeding mosquitoes on bacteria or on purified PGN. PGRPLB does not seem to regulate *Cec1* expression in the midgut in response to oral infections, which is in line with the study of Gendrin *et al* who reported an absence of control over *Cec1* in sugar and blood-fed mosquito midguts [29]. These results contrast with the role of PGRP-LB in *Drosophila* where it negatively regulates the Imd pathway in both systemic and local immune responses [30,31]. *PGRPLB* knockout flies showed prolonged (till day 4) *Diptericin* gene expression after septic infections with Gram-negative bacteria containing DAP-type PGN [30], which we did not observe in our system. Importantly, while *Drosophila* PGRP-LB exists as cytosolic and secreted isoforms [32], the *An. gambiae* PGRPLB is predicted to be membrane bound which could possibly limit its accessibility to released PGN fragments. However, it is currently difficult to draw accurate conclusions on *An. gambiae* PGPRLB, first because it is not clear whether it functions as an amidase for DAP-type or Lys-type PGN, and second because its silencing efficiency was modest approximating 35%, similar to what has been previously reported [29].

The cleavage of Rel2-F in the fat body is much more efficient with live than heat-inactivated bacteria, suggesting that the mosquito Imd pathway is likely activated by peptidoglycan fragments that are released from dividing cells through the activity of bacterial enzymes, such as autolysins, or host enzymes, like lysozyme [46,47]. Vaz *et al* have shown that recombinant *Drosophila* PGRP-LC and -SA can also engage peptidoglycan on the surface of Gram-positive bacteria *in vitro* especially in the absence of wall teichoic acids [48]. The fact that polymeric PGN of both *E. coli* and *S. aureus* triggered Rel2-F cleavage and since heat-inactivation is likely damaging to PGN [49], we cannot exclude that heat-inactivation may have also disrupted the direct interaction of mosquito PGRPLC with peptidoglycan exposed on the surface of Gram-positive bacteria.

The observation that polymeric PGN from *S. aureus* and *E. coli* triggers Rel2-F cleavage in the fat body agrees with a previous report showing that recombinant PGRPLC1 and PGRPLC3 bind polymeric DAP-type PGN of *E. coli* [26]. It is also possible that the activity of lysozyme on the injected polymeric PGN generated muropeptides that activated PGRPLC. Unfortunately, we were not able to secure muropeptides to compare their effects on Rel2-F cleavage to that of polymeric PGN.

In conclusion, our findings contribute to better understanding of the molecular make-up of the *An. gambiae* Imd pathway that plays an essential role in resistance to malaria parasites. A more refined understanding of immune pathways in vectors of disease will better guide genetic efforts to engineer parasite-refractory genotypes.

## Materials and methods

### Ethics statement

This study was carried out according to the recommendations in the Guide for the Care and Use of Laboratory Animals of the National Institutes of Health (Bethesda, USA). The animal protocol was approved by the Institutional Animal Care and Use Committee IACUC of the American University of Beirut (permit number 24-06-630). The IACUC complies with the Public Health Service Policy on the Humane Care and Use of Laboratory Animals (USA) and adopts the Guide for the Care and Use of Laboratory Animals of the National Institutes of Health.

### Rearing of *An. gambiae* mosquitoes

All experiments were performed on adult female *Anopheles gambiae* G3 strain mosquitoes. Mosquitoes were maintained in our insectary at 27 °C (± 0.5) and 75% (± 5%) humidity, with a 12-hour day-night cycle. Adults were maintained on 10% sucrose and were fed on BALB-c mice anesthetized using a ketamine/xylazine mixture for egg laying.

### Double stranded RNA synthesis and gene silencing by RNAi

DNA amplification of target genes was performed using T7 flanked primers listed in S1 Table. Gene-specific double-stranded RNAs (dsRNAs) were synthesized from T7-tagged purified PCR amplicons using the T7 RiboMax Express Large Scale RNA production system (Promega) according to the manufacturer’s instructions and purified as previously described [50]. Gene silencing by RNAi was performed by injecting 1–3-days-old adult female mosquitoes anesthetized over CO_2_ with 69 nl of a 3.5 μg/μl dsRNA solution for single gene knockdowns, 138 nl of a 1:1 mixture of two dsRNAs at 3.5 μg/μl each for double gene knockdowns, or 138 nl of a 1:1:1 mixture of three dsRNAs at 5 μg/μl each for triple gene knockdowns. Injections were done using a Drummond Nanoject II nanoliter injector.

### Bacterial infections and proliferation assays

The bacteria used in this study include, *E. coli* (GFP-expressing, ampicillin-resistant; [51]), *S. marcescens* strain DB11 (RFP-expressing, gentamycin-resistant; [52])*, B. cereus* (GFP-expressing, erythromycin-resistant; Kind gift from Dr. Laure Chamy, USJ)*, S. aureus* (GFP-expressing, chloramphenicol-resistant; [53]) and *E. faecalis* (kind gift from Dr. Antoine Abou Fayad, AUB Medical School). Bacteria were cultured overnight, washed and resuspended in sterile 1x phosphate-buffered saline (PBS) before use. Septic infections were performed by intrathoracic microinjection of mosquitoes with 69 nL of live bacterial suspensions at varying OD_600_ values (as specified in figure legends). Bacterial infections were performed 3 days post dsRNA-injection into mosquitoes. For infections using heat-killed bacteria, bacterial suspensions were incubated at 70°C for 10 minutes, and successful inactivation was confirmed by plating on Luria-Bertani (LB) agar supplemented with the appropriate antibiotic. For oral infections, mosquitoes were fed on sugar pads containing 3% sucrose solution and *S. marcescens* (OD_600_ = 15). Mosquitoes were starved for 8 hours before feeding on a 3% sucrose solution (control) or on *S. marcescens*-containing sugar pads for 12 hours. A food colorant was added to the pads to aid in sorting fed mosquitoes, which were then used in subsequent experiments. To assess mosquito resistance to bacterial infections, bacterial proliferation was measured in whole mosquito homogenates. Briefly, at designated time points post-infection, batches each of six bacteria-infected mosquitoes per genotype were homogenized in 500 µl LB broth. The homogenates were serially diluted, and 10 µl from each dilution was plated onto LB agar containing the appropriate antibiotic or on Selective Enterococcus Agar for *E. faecalis*. After overnight incubation at 37°C, bacterial Colony-Forming Units (CFUs) were quantified under a fluorescent stereoscope. All raw CFU data are listed in S2 Table.

### Western blot analysis

To assess the cleavage profile of Rel2-F in the mosquito fat body, abdomens (excluding the guts and the ovaries) were dissected in sterile 1x PBS from wild-type or dsRNA-treated adult female mosquitoes at different time points post-injection with *E. coli* (OD_600_ = 3), *S. marcescens* (OD_600_ = 1), *B. cereus* (OD_600_ = 2), *S. aureus* (OD_600_ = 3) or *E. faecalis* (OD_600_ = 3), from blood-fed mosquitoes, or from mosquitoes injected with pure peptidoglycan from *S. aureus* (Invivogen, cat# tlrl-pgns2)) or *E. coli* (Invivogen, cat# tlrl-kipgn) resuspended in endotoxin-free water at concentrations of 2 and 4 mg/ml. Abdomens were dissected from 6 mosquitoes per sample, homogenized in 60 μl of 1x Laemmli buffer for 1 min using a micro-pestle to lyse fat body cells, then centrifuged at 13000 g for 10 minutes at 4°C. Supernatants were collected into new tubes and 20 μl of each protein extract (equivalent to 2 abdomens) was supplemented with 5% β-*Mercaptoethanol,* boiled for 3 min and resolved by SDS-PAGE.

To assess the cleavage profile of Rel2-F in mosquito midguts (including cardia, anterior and posterior midgut), a group of 6 dsRNA-treated adult female mosquitoes fed with 3% sugar or *S. marcescens*-containing sugar pads were dissected in cold sterile 1x PBS supplemented with 1x protease inhibitor (PI) cocktail (Roche), then homogenized using micro-pestle in 30 μl of 1x Laemmli buffer containing PI for 30 secs. Samples were immediately boiled for 5 min to inhibit gut proteases, then centrifuged at 13000 g for 10 minutes at 4°C. Supernatants were collected into new tubes, supplemented with 5% β-*Mercaptoethanol* and resolved by SDS-PAGE.

All protein extracts were resolved using 8–10% gradient SDS-PAGE and wet transferred into immunoblot PVDF membranes using the BioRad Mini Trans-Blot Cell. Membranes were blocked with 1x PBS containing 0.05% Tween 20 and 3% milk for 1 hour at room temperature, followed by an overnight incubation at 4°C with the primary antibodies rabbit αRel2 (1:1000; produced by BOSTER) and mouse αActin (1:3000). Anti-rabbit or anti-mouse IgG horseradish peroxidase-conjugated secondary antibodies were used at 1:14000 and 1:6000 dilutions, respectively. Western blots were revealed with BioRad Clarity Max western ECL substrate and imaged using ChemiDoc MP (BioRad). Where indicated, band quantification was performed using Image Lab software. The raw data for the normalized Rel2 p90/p135 band densities are listed in S3 Table.

### RNA extraction and Real-Time PCR

Total RNA was extracted from 15 mosquito abdomens (excluding guts and ovaries) and 25 mosquito midguts (including cardia, anterior and posterior midguts) to assess *Cec1* and *Def1* gene expression in fat body and midguts, respectively, at the designated time points. To determine gene silencing efficiency, total RNA was extracted from 15 whole mosquitoes per gene knockdown at 3 days post-dsRNA injection. All tissues were collected and homogenized in TRIzol reagent (Invitrogen) and total RNA was extracted and purified as previously described [50]. First-strand cDNA was produced from 0.5-1 μg of total RNA using the iScript cDNA synthesis kit (Bio-Rad). QRT-PCR was performed in a CFX96 real-time detection system (Bio-Rad) using SYBR Green JumpStart Taq Ready Mix (Sigma-Aldrich) according to the manufacturer’s instructions. The *An. gambiae* ribosomal S7 gene was used as an endogenous control for normalization, and relative gene expression values were calculated using the comparative C_T_ method. Primers used in qRT-PCR are listed in S1 Table. All raw gene expression data are listed in S4 Table.

### Amplification of the 3′ end of Rel2-S transcript

The 3′ end of *Rel2-S* cDNA was amplified using the 3′ RACE system for rapid amplification of cDNA ends (Invitrogen) according to the manufacturer’s instructions. Briefly, total RNA was extracted from the abdomens of 15 *S. aureus*-infected mosquitoes at 6 hpi using TRIzol reagent (Invitrogen) according to the manufacturer’s instructions. Contaminant genomic DNA was removed by treatment with the RNAse-free DNAse I (Thermo Scientific). cDNA synthesis was performed using an oligo-dT containing adapter primer, as described by the RACE kit. The 3′ ends of *Rel2-F* and *Rel2-S* transcripts were amplified by PCR using a *Rel2*-specific primer (5′-GCCATTCCGGAAGGTCAAGA-3′) that anneals to the shared *RHD* sequence of both transcripts and a universal amplification primer that anneals to the oligo-dT containing adaptor primer (provided by the kit). The PCR generated 2 main amplicons, one at ~1800 bp which corresponds indeed to the size expected for Rel2-F and a second of ~ 600 bp that corresponds to Rel2-S (S6 Fig). The two *Rel2* amplicons were gel-purified, cloned into the pGEM-T and approximately 600 bp were sequenced from their 3′ end to identify potential sequences unique to Rel2-S.

## Supporting information

S1 Table List of primers used for dsRNA synthesis and qRT-PCR. (DOCX)

S2 Table Raw LOG2 transformed data of all CFU assays. (XLSX)

S3 Table Raw data of all the normalized p90/p135 band densities. (XLSX)

S4 Table Raw LOG2 transformed data of all *Cec1* and *Def1* expression analysis. (XLSX)

S1 Fig Alignment of the partial sequences of Rel2-S and Rel2-F. The last 3′ terminal 606 nucleotides of Rel2-S identified by 3′ RACE (including the 3′UTR) were aligned with their corresponding region in Rel2-F (spanning exons 5 (partially), 6 and 7). In bold are the 130 nucleotides at the distal 3′ end of Rel2-S that are unique to this transcript, of which 46 nucleotides are coding while the rest are in 3′ UTR. The stop codon in Rel2-S is shown in red.
(TIF)

S2 Fig Silencing Rel2 and Rel2-F compromises *S. aureus*-induced *Defensin 1* expression in the fat body. *Defensin* 1 (*Def1*) expression measured by qRT-PCR in the fat body of the indicated mosquito genotypes after injection with *S. aureus* (OD_600_ = 3) or sterile PBS (control). LOG2 transformed data are presented as mean ± SEM from 6 independent experiments (shown in different colors). Statistical analysis was performed using the two-tailed Mann-Whitney. **, *P* < 0.01.
(TIF)

S3 Fig PGRPLC and PGRPLB gene silencing efficiencies in sugar-fed mosquitoes. **(A)** The relative expression of the shown PGRPLC isoforms and of PGRPLB in single gene knockdowns was scored in sugar-fed whole mosquitoes at day 3 post-injection of the respective dsRNA and compared to the basal level expression in ds*LacZ*-injected control mosquitoes. Data shown are from 6 (PGRPLC1), 5 (PGRPLC2, PGRPLC3), and 4 (PGRPLB) independent biological experiments. (**B**) Relative expressions of PGRPLC1, PGRPLC2 and PGRPLC3 in sugar-fed whole mosquitoes at day 3 post-injection of ds*PGRPLC* (that targets a common exon in all PGRPLC splice variants) or a dsRNA mixture of ds*PGRPLC1*, ds*PGRPLC2* and ds*PGRPLC3*. Data shown are from at least 4 independent biological experiments. Shown are mean values (± SEM).
(TIF)

S4 Fig PGRPLB does not regulate Rel2 activation in the fat body during systemic bacterial infections. **(A)** *Cecropin 1* (*Cec1*) expression measured by qRT-PCR in the fat body of the indicated mosquito genotypes at 72 hpi with *S. aureus* (OD_600_ = 3) or injection of sterile PBS (control). Data are represented as mean ± SEM from 5 independent experiments (shown in different colors). Statistical analysis was performed using One-way ANOVA followed by Dunnett’s multiple comparison test. ***, *P* < 0.001. (**B**-**C**) Western blot analysis showing Rel2-F cleavage in the indicated gene knockdowns and times post-infection with (**B**) *S. aureus* (OD_600_ = 3) and (**C**) *E. coli* (OD_600_ = 3). β-actin was used as loading control. Each lane contains fat body extracts equivalent to 2 mosquito abdomens (excluding gut and ovaries). Red triangles indicate Rel2-p135 and Rel2-p90. The bar graphs in panels B and C represent the normalized p90/p135 band density from 4 and 3 independent experiments (shown in different colors), respectively. Statistical analysis was performed using the two-tailed Welch’s t-test. ns, non-significant.
(TIF)

S5 Fig Blood feeding does not activate Rel2-F in the fat body. Western blot analysis showing Rel2-F cleavage in the indicated gene knockdowns at (**A**) 6 hours, (**B**) 15 hours, and (**C**) 24 hours post-blood feeding. β-actin was used as loading control. Each lane contains fat body extracts equivalent to 2 mosquito abdomens (excluding gut and ovaries). Each image is representative of 2 independent experiments. Red triangles indicate Rel2-p135 and Rel2-p90.
(TIF)

S6 Fig Identification of the 3′ end of Rel2-S transcript by 3′ RACE. (**A**) Strategy of amplification of the 3′ end of Rel2-S transcript. UAP; universal amplification primer. (**B**) cDNA generated by 3′ RACE from total RNA isolated from the abdomens of naïve or *S. aureus* (OD_600_ = 3) infected mosquitoes.
(TIF)
