# Supplementary material for: A PGRPLC1/Rel2-F axis controls Anopheles gambiae resistance to systemic infections with Gram-positive bacteria containing Lys-type peptidoglycan
Source: PLoS Pathog. 2025 Sep 19;21(9):e1013527. doi: 10.1371/journal.ppat.1013527 (PMC12459809; doi:10.1371/journal.ppat.1013527)
Supplement: S1 Table — (DOCX) [file ppat.1013527.s001.docx]

| **S1_Table. List of primers used for dsRNA synthesis and qRT-PCR** | | |
| --- | --- | --- |
| **Gene** | **Primers used for dsRNA synthesis (T7 promoter sequence underlined; 5'-3')** | **Reference** |
| **LacZ** | For: TAATACGACTCACTATAGGGAGAATCCGACGGGTTGTTACT  Rev: TAATACGACTCACTATAGGGCACCACGCTCATCGATAATTT | [1] |
| **Rel2**  (AGAP006747) | For: TAATACGACTCACTATAGGGCGGAGAAGTCGAAGAAAACG  Rev: TAATACGACTCACTATAGGGCACAGGCACACCTGATTGAG | [2] |
| **Rel2-F** | For: TAATACGACTCACTATAGGGAATCCGACGCAACGATACG  Rev: TAATACGACTCACTATAGGGGACCGCAATGTGAAGGATG | [2] |
| **Rel2-S** | For: TAATACGACTCACTATAGGGTCAGCAGCAGCTCAAGTTCG  Rev: TAATACGACTCACTATAGGGAACCATTTGAAACAAATAATTTA |  |
| **PGRPLC1**  (AGAP005203-RC/RF) | For:taatacgactcactatagggCGCGGATGGCTTAAAGTTGG  Rev: taatacgactcactatagggCGCACAGCTTGTACTCCTTG | [3] |
| **PGRPLC2**  (AGAP005203-RD/RE) | For: taatacgactcactatagggGTTACAATTCCCGCAGCATAA  Rev: taatacgactcactatagggCGAGGCATATATTCGGTAACCT | [3] |
| **PGRPLC3**  (AGAP005203-RA/RB) | For: taatacgactcactatagggTTGATCCAGGAGTTTCATTCG  Rev: taatacgactcactatagggGAATGTTCCAATGAACGCGA | [3] |
| P**GRPLB**  (AGAP001212) | For: TaatacgactcactatagggAGACCATCCCGTACGTCATCATA  Rev: taatacgactcactatagggAGAATGTTCTTCGGTGGCAAATC | [4] |
| **PGRPLA1**  (AGAP005205-RA/RB) | For: TAATACGACTCACTATAGGGAGACCGACATTCCAAGCAACTTT  Rev: TAATACGACTCACTATAGGGAGAACCAGCCTAGCGTACAGCAT | [4] |
| **PGRPLA2**  (AGAP005205-RC) | For: TAATACGACTCACTATAGGGAGATGCTGATAACGCACATAGGC  Rev: TAATACGACTCACTATAGGGAGAGTTTCTCGCCGAACGTCAAT | [4] |
| **Gene** | **Primers used for qRT-PCR (5'-3')** | **Reference** |
| **S7**  (AGAP010592) | For: AGAACCAGCAGACCACCATC  Rev: GCTGCAA ACTTCGGCTATTC | [5] |
| **Cecropin 1**  (AGAP000693) | For: TCATCTTTGTCGTGCTGGCA  Rev : TCTTCAGCCGTCCCGCT | [4] |
| **Rel2** | For: GCCATTCCGGAAGGTCAAGA  Rev: AATGTCCGGATGATGGGCTGA |  |
| **Rel2-F** | For: AACCGAGCCCGATACGCTAC  Rev: GCCTGCTCCCACACGGTAAA |  |
| **Rel2-S** | For: GCAGGGCGAAATGTTGCGCAAG  Rev: CTCCATTGCTGAGGTTCGAACTT |  |
| **PGRPLC1** | For: CCGGTTAATAACGTCATCATTGC  Rev: TCACCGTAGTTGGTGTGGTT | [6] |
| **PGRPLC2** | For: CCGGTTAATAACGTCATCATTGC  Rev: CGTCGTAGTTTTTGCCATCC | [6] |
| **PGRPLC3** | For: CCGGTTAATAACGTCATCATTGC  Rev: TCGGGACTCGAATGAAACTC | [6] |
| **PGRPLB** | For: GTTGCGGATTTGCCACCGAA  Rev: ACATTCGGTCGTGCGTACCT |  |
| **PGRPLA1** | For: CTGGTTAGACTATCCCGGTG  Rev: GAATTCCGTTCCGCTCATTGA |  |
| **PGRPLA2** | For: GTCGCACATCGACAGGCACG  Rev: ACCTGGTAGTCCGCACGGGT |  |

**References**

1. Habtewold T, Povelones M, Blagborough AM, Christophides GK. Transmission blocking immunity in the malaria non-vector mosquito *Anopheles quadriannulatus* species A. PLoS Pathog. 2008;4(5):e1000070.

2. Meister S, Kanzok SM, Zheng XL, Luna C, Li TR, Hoa NT, et al. Immune signaling pathways regulating bacterial and malaria parasite infection of the mosquito *Anopheles gambiae*. Proc Natl Acad Sci U S A. 2005;102(32):11420-5.

3. Rodgers FH, Cai JA, Pitaluga AN, Mengin-Lecreulx D, Gendrin M, Christophides GK. Functional analysis of the three major PGRPLC isoforms in the midgut of the malaria mosquito Anopheles coluzzii. Insect Biochem Mol Biol. 2020;118:103288.

4. Gendrin M, Turlure F, Rodgers FH, Cohuet A, Morlais I, Christophides GK. The Peptidoglycan Recognition Proteins PGRPLA and PGRPLB Regulate Anopheles Immunity to Bacteria and Affect Infection by Plasmodium. J Innate Immun. 2017;9(4):333-42.

5. Garver LS, de Almeida Oliveira G, Barillas-Mury C. The JNK pathway is a key mediator of Anopheles gambiae antiplasmodial immunity. PLoS Pathog. 2013;9(9):e1003622.

6. Meister S, Agianian B, Turlure F, Relogio A, Morlais I, Kafatos FC, et al. *Anopheles gambiae* PGRPLC-mediated defense against bacteria modulates infections with malaria parasites. PLoS Pathog. 2009;5(8):e1000542.
