# Supplementary figures and images for: A PGRPLC1/Rel2-F axis controls Anopheles gambiae resistance to systemic infections with Gram-positive bacteria containing Lys-type peptidoglycan
Source: PLoS Pathog. 2025 Sep 19;21(9):e1013527. doi: 10.1371/journal.ppat.1013527 (PMC12459809; doi:10.1371/journal.ppat.1013527)

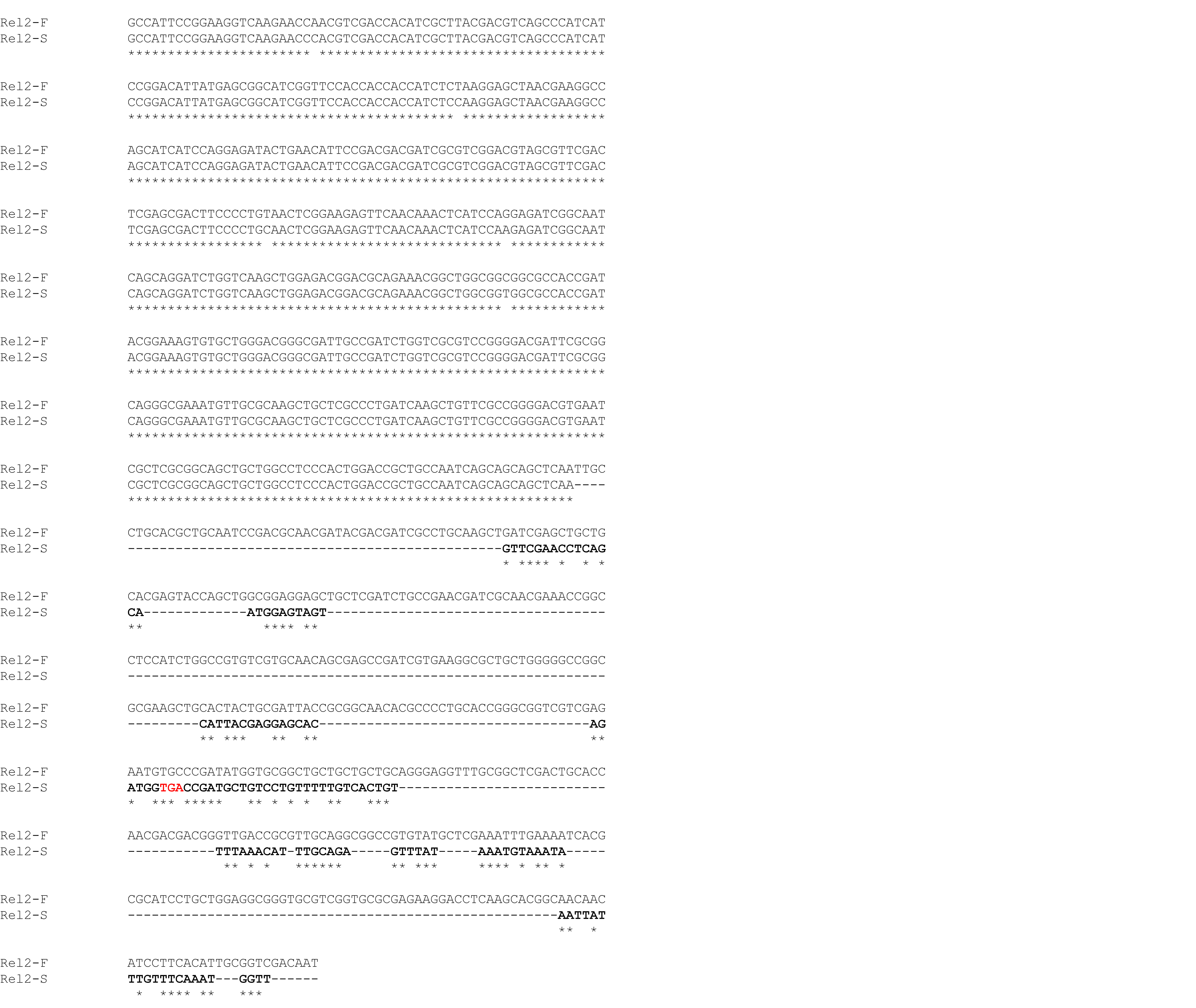

Supplement: S1 Fig — The last 3′ terminal 606 nucleotides of Rel2-S identified by 3′ RACE (including the 3′UTR) were aligned with their corresponding region in Rel2-F (spanning exons 5 (partially), 6 and 7). In bold are the 130 nucleotides at the distal 3′ end of Rel2-S that are unique to this transcript, of which 46 nucleotides are coding while the rest are in 3′ UTR. The stop codon in Rel2-S is shown in red. (TIF) [file ppat.1013527.s005.tif]

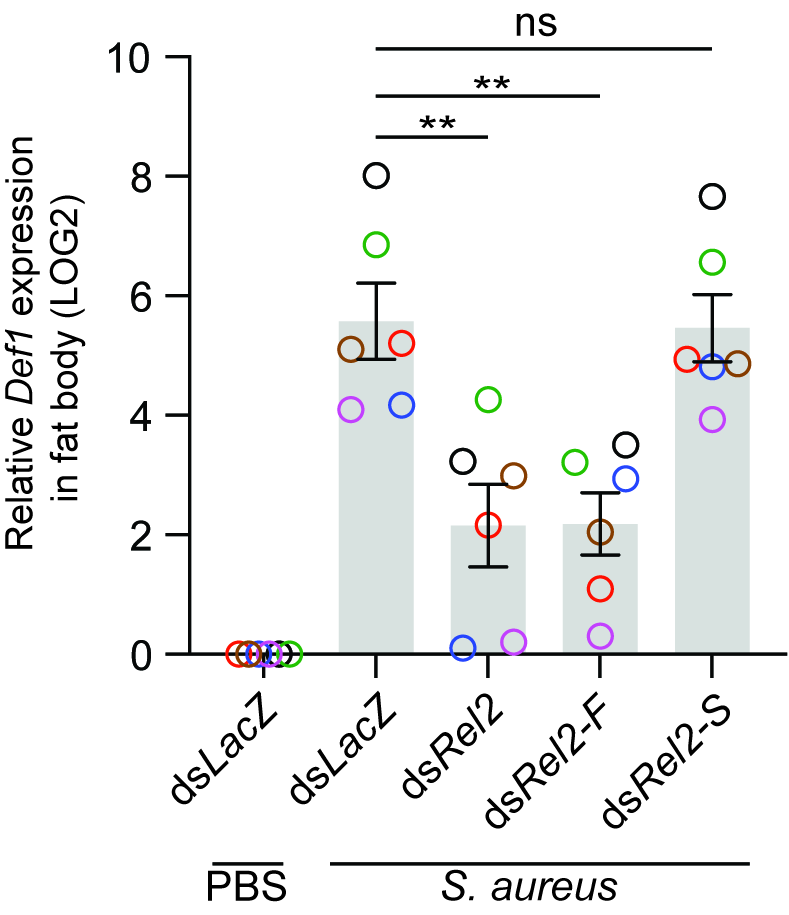

Supplement: S2 Fig — Defensin 1 (Def1) expression measured by qRT-PCR in the fat body of the indicated mosquito genotypes after injection with S. aureus (OD600 = 3) or sterile PBS (control). LOG2 transformed data are presented as mean ± SEM from 6 independent experiments (shown in different colors). Statistical analysis was performed using the two-tailed Mann-Whitney. **, P < 0.01. (TIF) [file ppat.1013527.s006.tif]

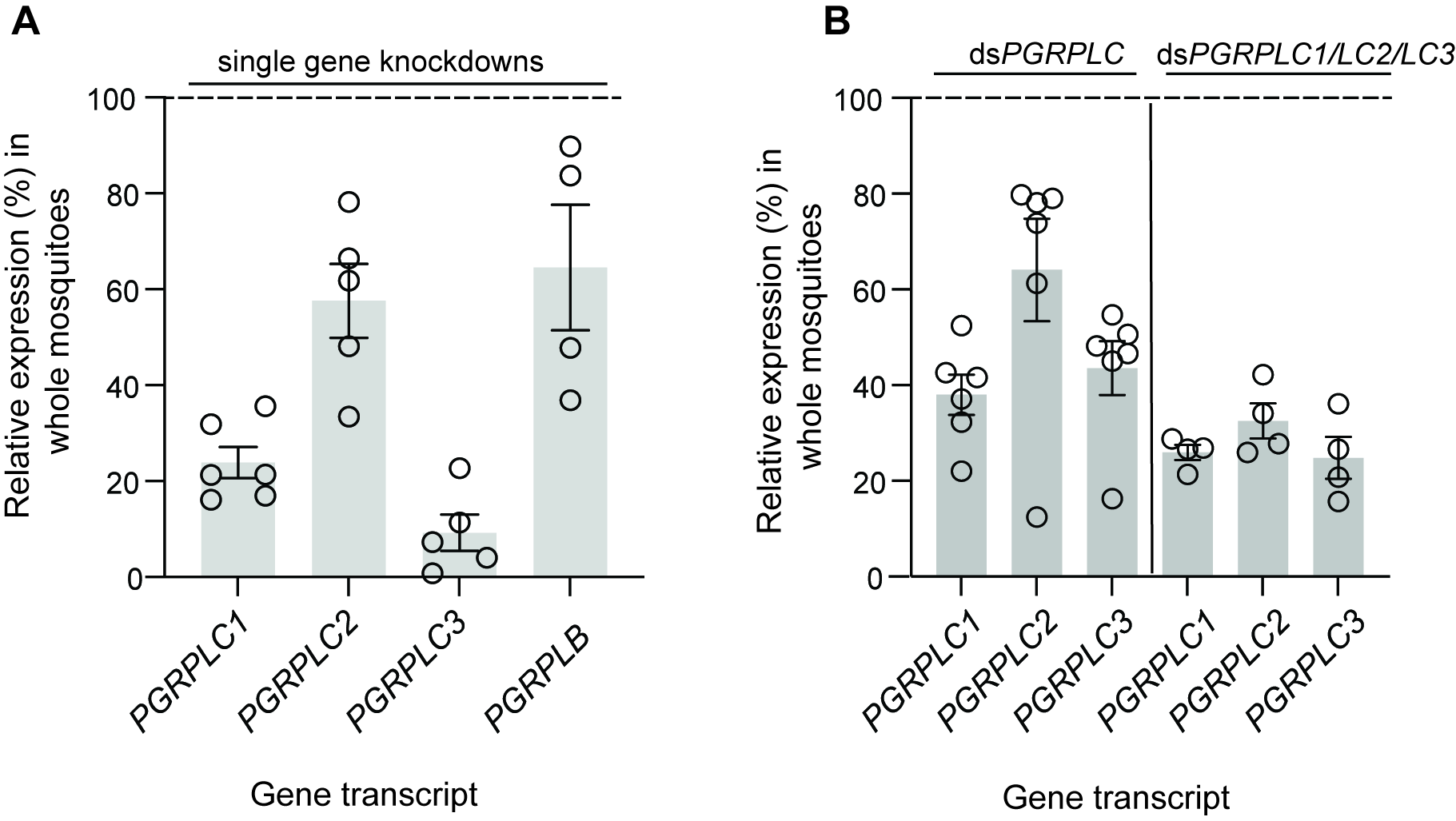

Supplement: S3 Fig — (A) The relative expression of the shown PGRPLC isoforms and of PGRPLB in single gene knockdowns was scored in sugar-fed whole mosquitoes at day 3 post-injection of the respective dsRNA and compared to the basal level expression in dsLacZ-injected control mosquitoes. Data shown are from 6 (PGRPLC1), 5 (PGRPLC2, PGRPLC3), and 4 (PGRPLB) independent biological experiments. (B) Relative expressions of PGRPLC1, PGRPLC2 and PGRPLC3 in sugar-fed whole mosquitoes at day 3 post-injection of dsPGRPLC (that targets a common exon in all PGRPLC splice variants) or a dsRNA mixture of dsPGRPLC1, dsPGRPLC2 and dsPGRPLC3. Data shown are from at least 4 independent biological experiments. Shown are mean values (± SEM). (TIF) [file ppat.1013527.s007.tif]

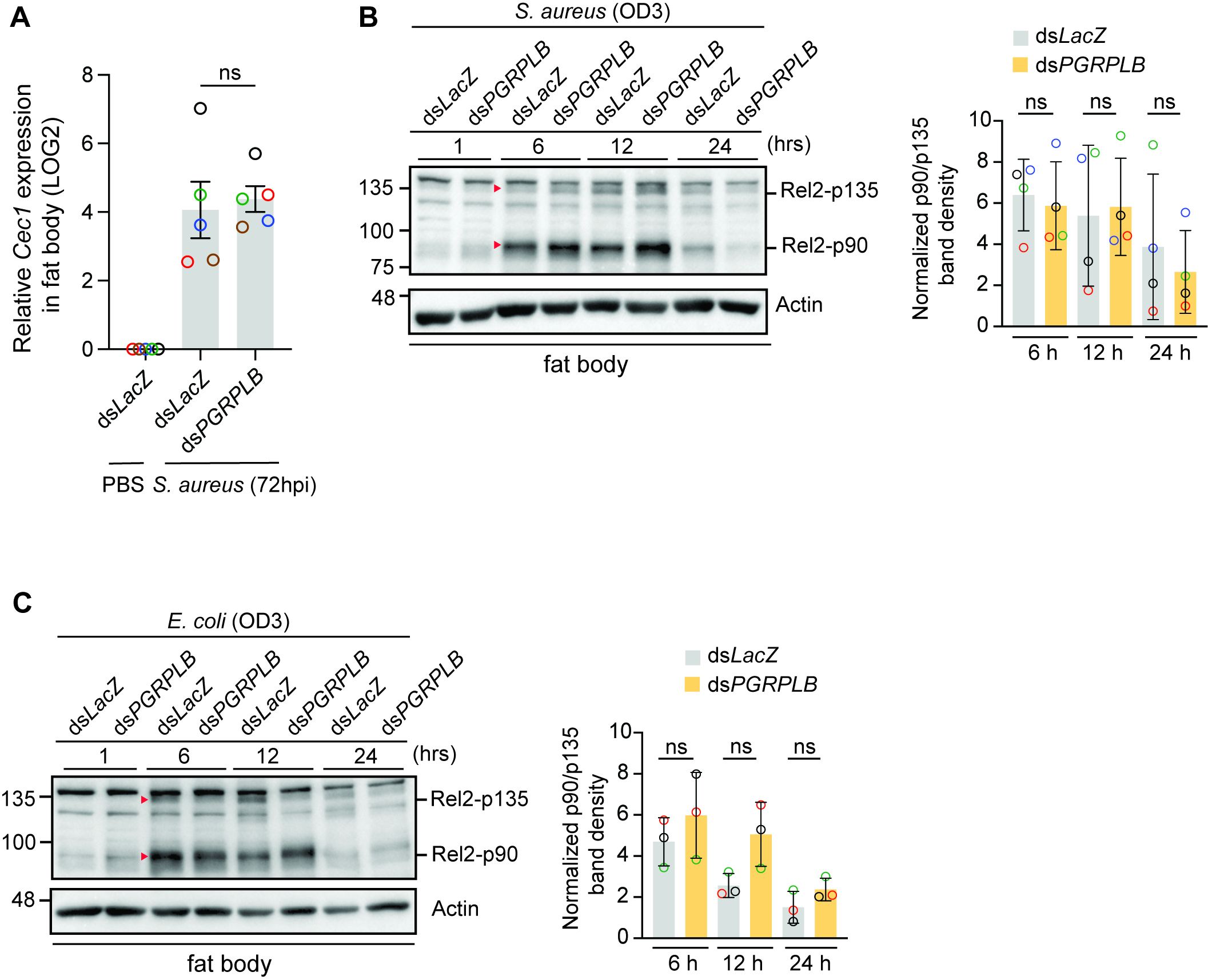

Supplement: S4 Fig — (A) Cecropin 1 (Cec1) expression measured by qRT-PCR in the fat body of the indicated mosquito genotypes at 72 hpi with S. aureus (OD600 = 3) or injection of sterile PBS (control). Data are represented as mean ± SEM from 5 independent experiments (shown in different colors). Statistical analysis was performed using One-way ANOVA followed by Dunnett’s multiple comparison test. ***, P < 0.001. (B-C) Western blot analysis showing Rel2-F cleavage in the indicated gene knockdowns and times post-infection with (B) S. aureus (OD600 = 3) and (C) E. coli (OD600 = 3). β-actin was used as loading control. Each lane contains fat body extracts equivalent to 2 mosquito abdomens (excluding gut and ovaries). Red triangles indicate Rel2-p135 and Rel2-p90. The bar graphs in panels B and C represent the normalized p90/p135 band density from 4 and 3 independent experiments (shown in different colors), respectively. Statistical analysis was performed using the two-tailed Welch’s t-test. ns, non-significant. (TIF) [file ppat.1013527.s008.tif]

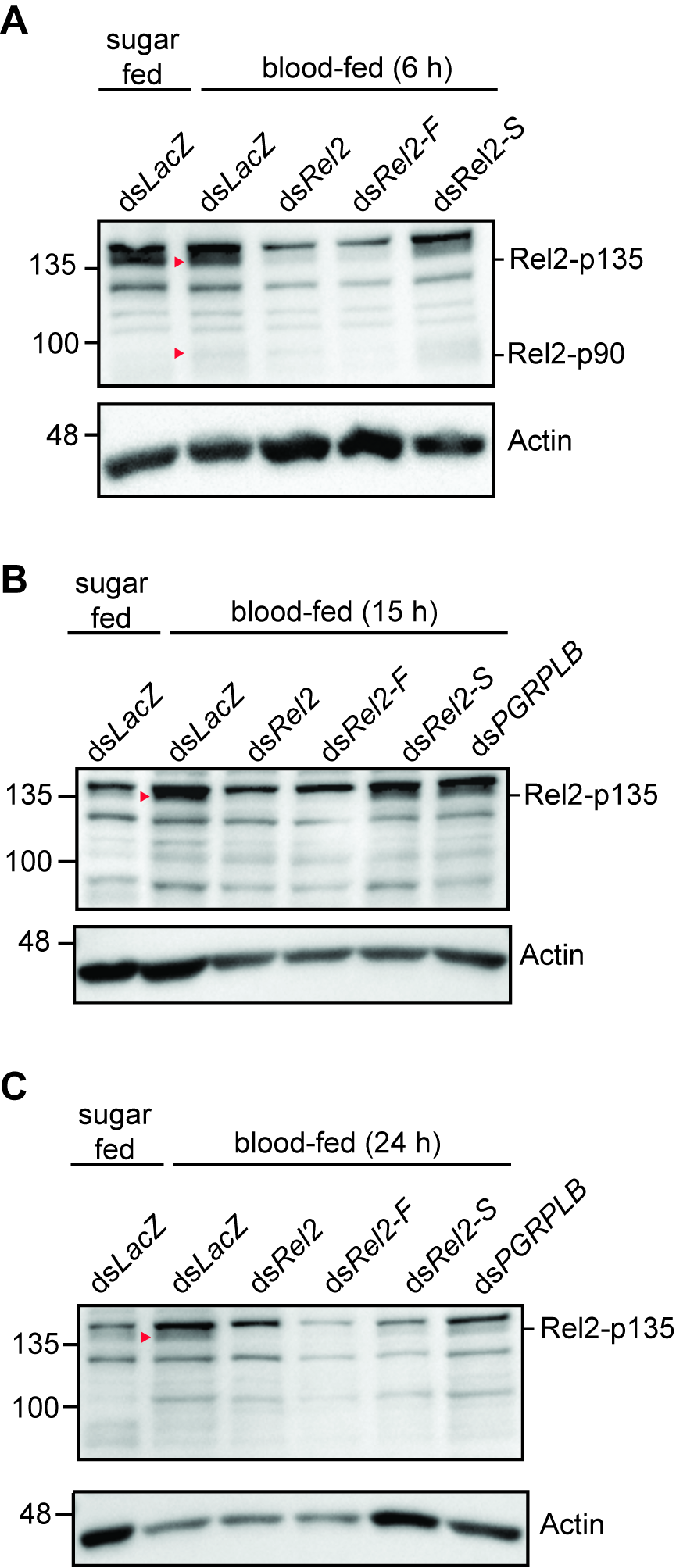

Supplement: S5 Fig — Western blot analysis showing Rel2-F cleavage in the indicated gene knockdowns at (A) 6 hours, (B) 15 hours, and (C) 24 hours post-blood feeding. β-actin was used as loading control. Each lane contains fat body extracts equivalent to 2 mosquito abdomens (excluding gut and ovaries). Each image is representative of 2 independent experiments. Red triangles indicate Rel2-p135 and Rel2-p90. (TIF) [file ppat.1013527.s009.tif]

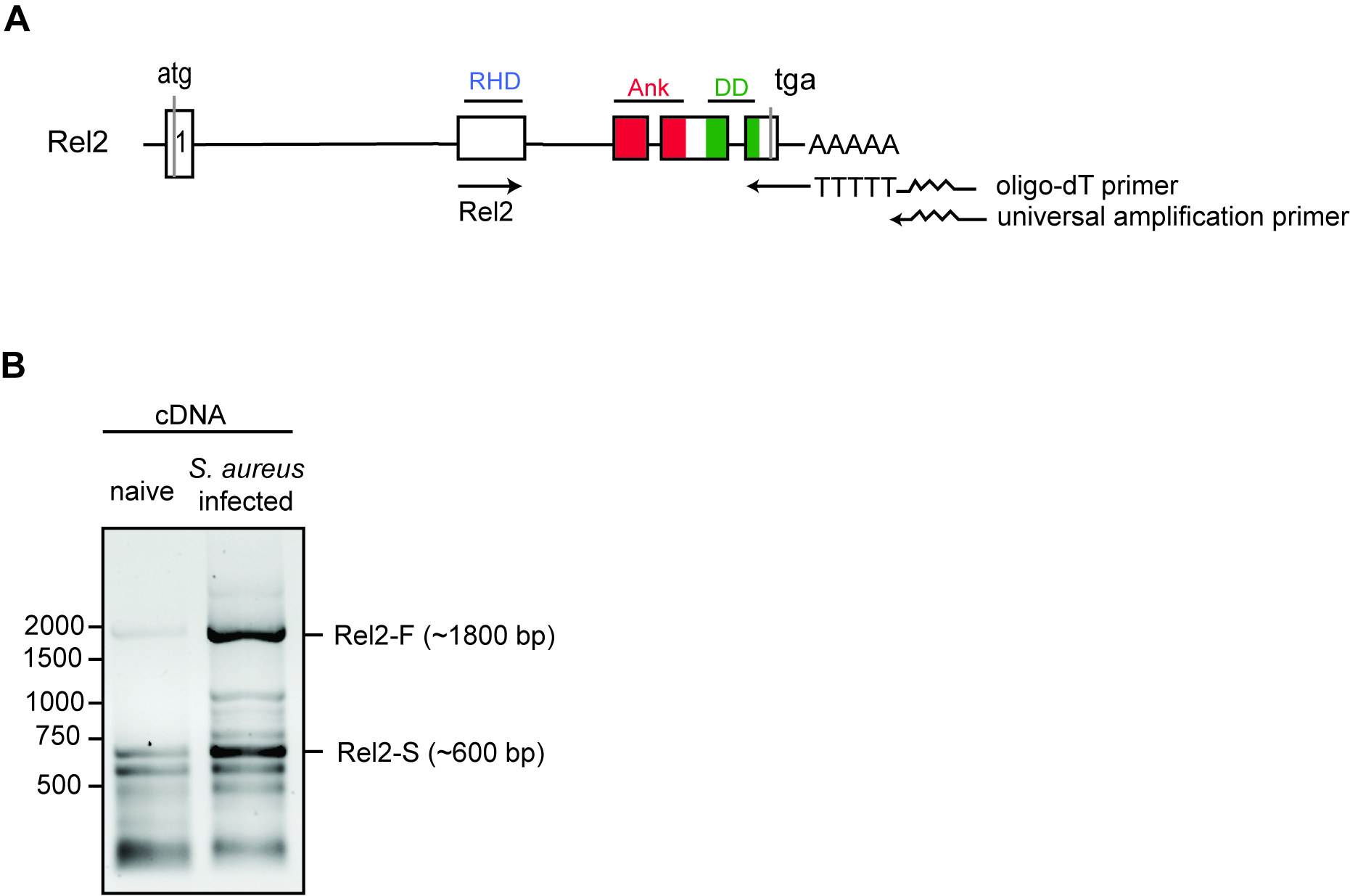

Supplement: S6 Fig — (A) Strategy of amplification of the 3′ end of Rel2-S transcript. UAP; universal amplification primer. (B) cDNA generated by 3′ RACE from total RNA isolated from the abdomens of naïve or S. aureus (OD600 = 3) infected mosquitoes. (TIF) [file ppat.1013527.s010.tif]
